# Carbohydrate-rich breakfast attenuates glycaemic, insulinaemic and ghrelin response to *ad libitum* lunch relative to morning fasting in lean adults

**DOI:** 10.1017/S0007114515001506

**Published:** 2015-05-25

**Authors:** Enhad A. Chowdhury, Judith D. Richardson, Kostas Tsintzas, Dylan Thompson, James A. Betts

**Affiliations:** 1 Department for Health, University of Bath, BathBA2 7AY, UK; 2 School of Life Sciences, Queen's Medical Centre, University of Nottingham, Nottingham, UK

**Keywords:** Breakfast skipping, Appetite hormones, Insulin sensitivity, Second-meal effect, Energy intake

## Abstract

Breakfast omission is associated with obesity and CVD/diabetes, but the acute effects of extended morning fasting upon subsequent energy intake and metabolic/hormonal responses have received less attention. In a randomised cross-over design, thirty-five lean men (*n* 14) and women (*n* 21) extended their overnight fast or ingested a typical carbohydrate-rich breakfast in quantities relative to RMR (i.e. 1963 (sd 238) kJ), before an *ad libitum* lunch 3 h later. Blood samples were obtained hourly throughout the day until 3 h post-lunch, with subjective appetite measures assessed. Lunch intake was greater following extended fasting (640 (sd 1042) kJ, *P*< 0·01) but incompletely compensated for the omitted breakfast, with total intake lower than the breakfast trial (3887 (sd 1326) *v.* 5213 (sd 1590) kJ, *P*< 0·001). Systemic concentrations of peptide tyrosine–tyrosine and leptin were greater during the afternoon following breakfast (both *P*< 0·05) but neither acylated/total ghrelin concentrations were suppressed by the *ad libitum* lunch in the breakfast trial, remaining greater than the morning fasting trial throughout the afternoon (all *P*< 0·05). Insulin concentrations were greater during the afternoon in the morning fasting trial (all *P*< 0·01). There were no differences between trials in subjective appetite during the afternoon. In conclusion, morning fasting caused incomplete energy compensation at an *ad libitum* lunch. Breakfast increased some anorectic hormones during the afternoon but paradoxically abolished ghrelin suppression by the second meal. Extending morning fasting until lunch altered subsequent metabolic and hormonal responses but without greater appetite during the afternoon. The present study clarifies the impact of acute breakfast omission and adds novel insights into second-meal metabolism.

Regular breakfast omission is associated with greater risk of obesity^(^ ^1^ ^,^ ^2^ ^)^, prospective weight gain^(^ ^3^ ^)^, type 2 diabetes^(^ ^4^ ^,^ ^5^ ^)^ and CHD^(^ ^6^ ^)^. Increased adiposity is due to chronic positive energy balance, so laboratory studies have understandably explored a possible role of daily breakfast in regulating energy intake (EI) and associated metabolic responses. However, the focus of previous research in adults has been to compare breakfasts of varied quantity or composition^(^ ^7^ ^–^ ^14^ ^)^. In contrast, the effects of a morning fasting condition *v.* breakfast consumption on EI and associated metabolic responses later in the same day has received less attention.

Laboratory studies in adults that have compared intake following breakfast omission have yielded equivocal results, with both reduced^(^ ^15^ ^,^ ^16^ ^)^ and similar^(^ ^17^ ^)^ overall EI observed. To the authors' knowledge, only two studies^(^ ^15^ ^,^ ^17^ ^)^ have measured hormonal and appetite responses to breakfast omission/consumption followed by an *ad libitum* lunch. These studies therefore provide valuable information, but their designs included mid-morning preloads (i.e. a standardised feeding of 1050 kJ (250 kcal) and 1500 kJ (358 kcal) in all trials before lunch) such that the effects of unbroken overnight fasting prior to an *ad libitum* lunch were not a focus. However, there is evidence in adolescent girls that breakfast consumption can reduce hunger relative to morning fasting, with high protein breakfasts reducing daily ghrelin (an appetite-stimulating hormone^(^ ^18^ ^)^), increasing peptide tyrosine–tyrosine^(^ ^19^ ^)^ (PYY; a hormone associated with satiety^(^ ^20^ ^)^) and reducing *ad libitum* lunch intake^(^ ^21^ ^)^, but in both cases without reducing total EI. In addition, the two preload studies^(^ ^15^ ^,^ ^17^ ^)^ described in adults have demonstrated that breakfast omission prior to a preload can affect subsequent metabolic responses along with hormonal outcomes such as PYY and glucagon-like peptide-1^(^ ^17^ ^)^ (shown to augment insulin secretion to nutrients and reduce food intake^(^ ^22^ ^)^). Omission of breakfast therefore has the potential to affect EI as well as metabolic and hormonal responses to subsequent feeding.

The metabolic and hormonal responses to an *ad libitum* lunch when still in an overnight fasted state remain to be examined in adults. This question is of relevance both in terms of understanding the basic physiology influencing daily feeding patterns and in terms of practical relevance for the 19–28 % of Western societies who frequently skip breakfast altogether^(^ ^23^ ^,^ ^24^ ^)^. Accordingly, we examined acute EI, appetite-regulatory hormones and metabolic responses after extended morning fasting relative to a standardised high glycaemic index/carbohydrate breakfast. We hypothesised that the prescribed differences in EI during the morning would not be fully compensated for at lunch. Secondly, that morning fasting would result in greater appetite sensations consistent with increased orexigenic (ghrelin) and lower anorectic (e.g. PYY) hormone responses throughout the day.

## Methods

### Participants

The present study was part of a wider randomised controlled trial (the Bath Breakfast Project), other results from which have been published previously^(^ ^25^ ^)^, for which 301 individuals were invited for eligibility assessment, 231 were assessed for eligibility, 137 were invited to participate and thirty-eight agreed to do so (three dropped out prior to testing). Here we report data for thirty-five healthy, lean men (*n* 14) and women (*n* 21) aged 22–56 years who completed this part of the project^(^ ^26^ ^)^. Within the present study cohort, there was a mix of frequent habitual breakfast consumers (classified as >209 kJ (>50 kcal) intake within 2 h of waking on ≥ 4 d of the week; *n* 27) and infrequent consumers (*n* 8). The sample size for this investigation was determined by estimates for the wider project described previously^(^ ^26^ ^)^. In brief, those estimates were based on the statistical power required to detect differences in free-living diet and physical activity between two independent groups (*n* 14). The total sample size for the current investigation (*n* 35) is therefore more than adequate to confidently detect any meaningful within-subjects responses of the more tightly controlled laboratory-based measures assessed here using a repeated measures cross-over design. Participants were recruited via local advertisement from South West England and were initially assessed for eligibility based on a BMI of 18–25 kg/m^2^ and later classified as lean based on dual-energy X-ray absorptiometry-derived fat mass indices of ≤ 7·5 kg/m^2^ (men) and ≤ 11 kg/m^2^ (women)^(^ ^27^ ^)^. Recruitment and laboratory visits spanned from 10 June 2010 to 16 May 2013. In accordance with the full eligibility criteria set out previously^(^ ^26^ ^)^, participants reported being weight stable ( ± 1 kg body mass within past 6 months), were free of metabolic disorders and adhered to a standard sleep–wake cycle (e.g. no shift workers). Participant characteristics are presented in Table 1.

**Table 1:** Participant characteristics (Mean values and standard deviations, *n* 35)

Characteristics	Mean	sd
Age (years)	36	11
Body mass (kg)	67·9	9·2
BMI (kg/m^2^)	22·7	2·5
Fat mass index* (kg/m^2^)		
Female	6·7	2·0
Male	4·1	1·4
Habitual breakfast consumers† (*n*)	27	
Female (*n*)	21	

* Fat mass index calculated as dual-energy X-ray absorptiometry-derived total fat mass divided by height squared.

† Habitual breakfast consumption defined as >209 kJ (>50 kcal) intake within 2 h of waking on ≥ 4 d/week.

### Ethical approval

The present study was conducted according to the guidelines laid down in the Declaration of Helsinki and all procedures involving human subjects were approved by the National Health Service South West 3 Research Ethics Committee. Written informed consent was obtained from all participants. The present study is registered with Current Controlled Trials (http://www.isrctn.com) (ISRCTN31521726).

### Study design

Each participant undertook a randomised, counterbalanced cross-over study design involving two laboratory-based feeding trials in the Human Physiology Laboratories at the University of Bath. Prior to their first day in the laboratory, participants recorded a 48-h weighed food and drink intake diary and repeated this prior to their second visit. In the 24 h prior to each laboratory visit, participants abstained from strenuous exercise, as well as caffeine and alcohol intake. These trials were separated by 3–28 d where participants resumed their habitual routine, with eumenorrheic women undergoing testing during the follicular phase of the menstrual cycle (3–10 d after onset of menses)^(^ ^28^ ^,^ ^29^ ^)^. Dual-energy X-ray absorptiometry scans to assess the body composition of these participants occurred at a separate visit following the two laboratory visits described here.

### Protocol for laboratory visits

On arrival at the laboratory at 08.00 hours ± 1 h, following an overnight fast of ≥ 10 h, participants voided and had body mass measured in light clothing (Seca 873; Vogel and Halke). RMR was assessed as described later, before a cannula was inserted into an antecubital vein, with a baseline sample of 15 ml blood obtained. Participants were then provided with either a breakfast (to be consumed within 15 min) or asked to rest for the same duration, with blood samples taken at 15 min, 30 min and an hour after completion of the breakfast period. Blood samples were then obtained hourly until 3 h post-breakfast, at which point an *ad libitum* lunch was provided. The lunch period was of 30-min duration with participants in isolation (i.e. all participants were tested entirely separately). Blood samples were obtained hourly from 1 h after completion of the lunch until 3 h after lunch. Participants also completed visual analogue scales relating to appetite throughout the day while remaining sedentary and completing quiet activities such as reading, watching television and typing. Participants remained in the laboratory throughout the duration of each testing visit.

### Breakfast

The breakfast consisted of corn flakes (Kellogg's), 2 % fat milk (Sainsbury's), toasted white bread (Braces), margarine (I can't believe it's not butter) and fresh orange juice (Sainsbury's) and was based on the breakfast provided by Chryssanthopoulos *et al.* ^(^ ^30^ ^)^. Participants were given the choice of white sugar added to cornflakes, or seedless raspberry jam (Sainsbury's) on their toast, or an isoenergetic combination of both. The overall percentages of energy from macronutrients in the breakfast were 70 % carbohydrate, 17 % fat and 13 % protein. This breakfast was selected because it is typical of the type of carbohydrate-rich foods commonly consumed at breakfast in the UK^(^ ^24^ ^)^ and also therefore provides an effective challenge to glycaemic control relative to the morning fasting trial. The breakfast was provided in quantities that contained 0·06 g carbohydrate per kcal of each individual participant's measured daily RMR, resulting in an EI of 1963 (sd 238) kJ (469 (sd 57) kcal). The size of this breakfast was based upon pilot work to determine an ecologically valid portion size, which also surpasses one of the most commonly accepted EI ranges of ‘breakfast’ as a minimum of 20 % of daily EI^(^ ^31^ ^)^, whilst also standardising for individual differences in RMR as it directly reflects energy requirements during our resting trial and is a primary driver of EI^(^ ^32^ ^)^. Participants were first provided with cereal, then at 5-min intervals, toast and finally orange juice, with all of the breakfast consumed within 15 min to standardise any effects of eating rate upon appetite hormones^(^ ^33^ ^)^. During the morning fasting trial, participants sat quietly for a matched time period. No food or drink was allowed following consumption of the breakfast or completion of the matched rest period until the *ad libitum* lunch; therefore, in the breakfast trial, the overnight fasting duration was ≥ 11 h and in the morning fasting trial was ≥ 14·5 h.

### Ad libitum lunch

Three hours post-breakfast, participants were provided with an *ad libitum* lunch test meal consisting of 1 kg cooked (i.e. wet weight) penne pasta (Sainsbury's) including tomato sauce (Ragu); prepared at a ratio of 1:1 uncooked mass. Each pasta meal was prepared according to specified cooking times, with the energy density of the cooked pasta calculated (to account for any differences in water absorption) and the final masses of pasta and sauce combined noted, in order to calculate the energy density for the homogeneous mixture of pasta and sauce. The overall percentages of energy from macronutrients for the lunch were 79 % carbohydrate, 14 % fat and 7 % protein. Consistent with the rationale for the carbohydrate-rich breakfast, this meal was selected on the basis that it would also challenge glycaemic control. Pasta was provided in a bowl which was replenished every 10 min to minimise visual feedback relating to consumed volume, and to prevent any tendency to finish the portion provided. Participants were left alone during the lunch with a recorded message played requesting participants eat until they had satisfied their hunger. During their first trial, participants were allowed *ad libitum* intake of plain water during lunch; this volume of water was subsequently replicated on their second visit.

### Expired gas analysis

Expired air samples were collected in 200 litre Douglas bags (Hans Rudolph) via falconia tubing (Baxter, Woodhouse and Taylor), with concurrent measurement of inspired air composition^(^ ^34^ ^)^. The obtained samples were passed through tubing containing anhydrous calcium sulphate (Drierite) to remove water content from the samples. Relative proportions of O_2_ and CO_2_ were measured in a known volume of the sample using paramagnetic and IR analysers, respectively (Servomex 1440). This analyser was calibrated on the morning of each testing day using two gases of known composition (British Oxygen Company). The volume of expired air was established using a dry gas meter (Harvard Apparatus), with the temperature of the expired gases measured using a CheckTemp1C (Hanna Instruments) during evacuation from the Douglas bags.

Samples for RMR were collected in accordance with guidelines for best practice by Compher *et al.* ^(^ ^35^ ^)^. Rates of both O_2_ utilisation (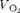) and CO_2_ production (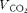) were used to calculate energy expenditure^(^ ^36^ ^)^ corrected for urinary N excretion^(^ ^37^ ^)^:
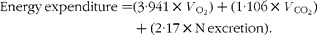



### Blood sampling

The intravenous cannula was kept patent through regular flushing with 0·9 % NaCl infusion, with the first 5 ml of each blood draw discarded. Whole blood was dispensed into tubes coated with EDTA for collection of plasma and immediately centrifuged. Serum was obtained by dispensing whole blood into a serum separation tube, which was left to stand at room temperature for 45 min before centrifugation. For analysis of acylated ghrelin, 1 ml of whole blood was dispensed into a tube coated with EDTA, which had 50 μl of a *p*-hydroxymercuribenzoic acid solution (prepared as a 100 mm concentrate solution in potassium phosphate buffer containing 1·2 % 10 m-NaOH)^(^ ^38^ ^)^. Samples were then centrifuged with 500 μl of the supernatant transferred to an untreated blood tube containing 10 μl 1 m-HCl. Samples were centrifuged again and the supernatants removed. Centrifugation was at 3466 ***g*** for 10 min at 4°C in all cases, with centrifuged samples immediately cooled using dry ice and then stored at − 80°C for subsequent analysis.

### Urine collection

Participants' urine was collected in a vessel containing 5 ml of 10 % thymol isopropanol, which acted as a preservative. For a given measurement period, the collected urine was mixed thoroughly, with a 1 ml aliquot obtained and kept at − 80°C prior to analysis. Analysis of urinary urea was conducted using a commercially available immunoassay as described later for plasma.

### Analysis of blood samples

Total (intra-assay CV, 4·0 %, inter-assay CV, 7·8 %) and acylated ghrelin (intra-assay CV, 4·2 %, inter-assay CV, 11·3 %) (Bertin Pharma), PYY (intra-assay CV, 4·3 %, inter-assay CV, 11·1 %) and total glucagon-like peptide-1 (intra-assay CV, 4·8 %, inter-assay CV, 27·0 %) (Merck Millipore) assays were conducted using plasma, with leptin (intra-assay CV, 3·4 %, inter-assay CV, 6·4 %) (R&D Systems, Inc.) and insulin (intra-assay CV, 4·7 %, inter-assay CV, 12·5 %) (Mercodia AB) assays conducted using serum. All assays were done using commercially available ELISA, employed according to manufacturer's instructions, with all samples batches analysed at the conclusion of the study and samples from each participant included on the same plate. Analysis of blood samples for NEFA (intra-assay CV, < 5 %, inter-assay CV, < 5 %), glucose (intra-assay CV, < 5 %, inter-assay CV, < 6 %) and urea (intra-assay CV, < 5 %, inter-assay CV, < 3 %) were conducted using plasma on a Daytona (Randox Laboratories) automated analyser according to manufacturer's instructions using commercially available immunoassays (Randox Laboratories).

### Appetite sensations

Paper-based, 100-mm visual analogue scales were used to assess subjective appetite. These measurements were obtained immediately pre- and post-breakfast, immediately pre- and post-lunch and after a 3-h postprandial period following lunch. Participants marked their response to questions assessing desire to eat, hunger, fullness and prospective consumption with anchor terms on each end of the scale (e.g. not at all hungry *v*. as hungry as I have ever felt). Higher scores were indicative of greater sensations. A composite appetite score^(^ ^39^ ^)^ was calculated as follows:
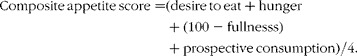



### Statistical analysis

For single comparisons of two means (e.g. EI at lunch), the distribution of all data was verified as normal using a Shapiro–Wilk test and paired *t* tests were thus employed throughout. For comparison of time series variables that were measured over the course of the day in each condition (e.g. appetite hormones), repeated measures ANOVA (breakfast/fasting × time point) were employed with Greenhouse–Geisser corrections applied to intra-individual contrasts for ɛ < 0·75, and the Huynh–Feldt correction applied for less severe asphericity^(^ ^40^ ^)^. Significant interactions were explored with multiple *t* tests to locate variance between trials at level time points, with a Holm–Bonferroni stepwise adjustment^(^ ^41^ ^)^. Statistical significance was accepted at *P*≤ 0·05. Data are presented in text as means and standard deviations, figures display mean with normalised CI. These CI represent the comparison between the two trials, removing the inter-individual variation due to the fully paired nature of the experimental design^(^ ^42^ ^)^. All statistical analyses were conducted using IBM SPSS statistics version 22 (IBM).

## Results

### Energy intake

**Fig. 1:**
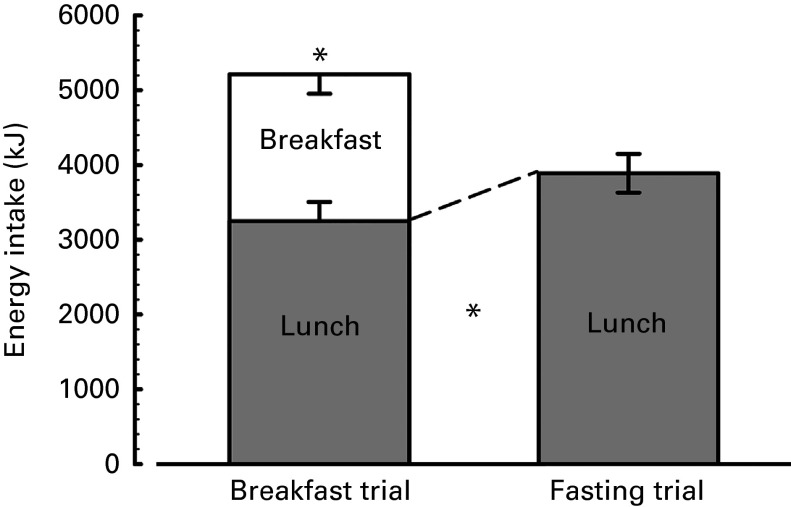
Energy intake during trials. In the morning fasting trial, an asymmetric normalised CI is plotted, the negative portion of which reflects the comparison between lunches and the positive portion reflects the comparison against total intake (i.e. lunch plus breakfast). An asterisk above a bar represents the comparison between the sum of the components of the bar, an asterisk between the bars represents the comparison between the specific component (*P*< 0·01). *n* 34, as one individual felt nauseous prior to lunch provision on one visit.

During the breakfast trial, participants consumed a prescribed breakfast of 1963 (sd 238) kJ (469 (sd 57) kcal) (i.e. variance proportionate to inter-individual differences in RMR). During the *ad libitum* lunch, participants consumed 3247 (sd 1460) kJ (776 (sd 349) kcal) following breakfast but significantly more in the morning fasting trial (3887 (sd 1326) kJ (929 (sd 317) kcal); *P*< 0·01, Fig. 1). However, when the absolute EI during the breakfast trial was calculated (breakfast+lunch), this was significantly more than during the morning fasting trial (5213 (sd 1590) *v.* 3887 (sd 1326) kJ (1246 (sd 380) *v.* 929 (sd 317) kcal); *P*< 0·001). When comparing the EI at lunch in the two conditions, the additional EI of 640 (sd 1042) kJ (153 (sd 249) kcal) during the morning fasting trial accounted for approximately 33 % of the prescribed breakfast (i.e. additional intake at lunch during the fasting trial was insufficient to compensate for the breakfast provided).

### Glucose

**Fig. 2:**
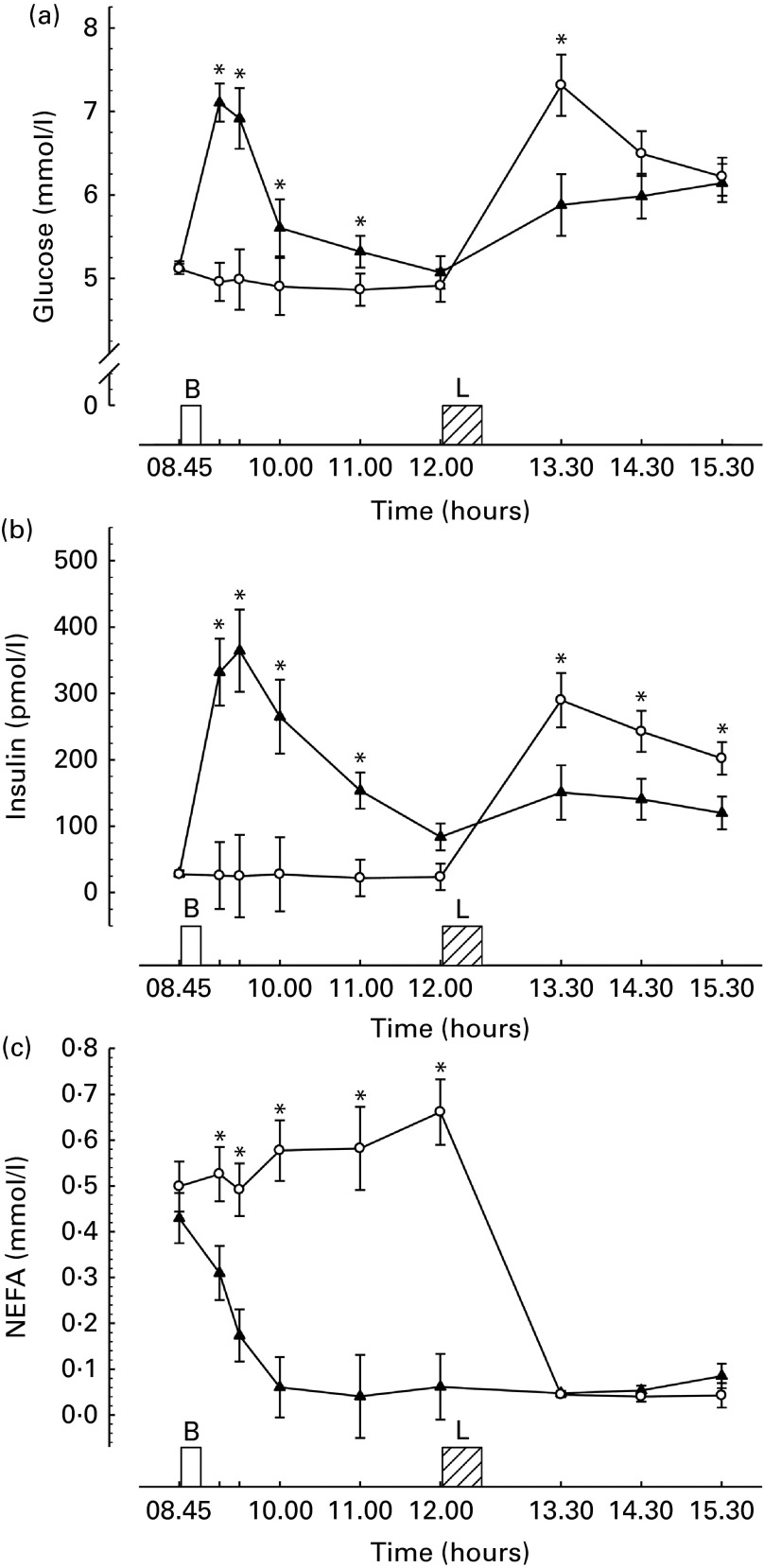
Metabolic responses during trials. (a) Plasma glucose (*n* 32), (b) serum insulin (*n* 32), (c) plasma NEFA (*n* 31), where missing data are due to insufficient blood for analysis. Values are means with their normalised CI represented by vertical bars. * Mean value was significantly different from the corresponding time point in other trial (*P*< 0·03). B, breakfast period, in which participants ate a prescribed breakfast during the breakfast trial and rested during the morning fasting trial. L, *ad libitum* pasta lunch. –▲–, Breakfast; –○–, fasting.

There were main effects of time, trial and a trial × time interaction for plasma glucose concentrations (all *P <*0·01). Glucose concentrations were significantly greater than fasting following breakfast consumption until 2 h post-breakfast (all *P <*0·03, Fig. 2(a)). By 3 h post-breakfast, there was no difference in plasma glucose concentrations between trials (*P*= 0·3). Glucose concentrations were significantly greater 1 h post-lunch in the morning fasting trial (*P <*0·01). There was a strong tendency for greater concentrations in the morning fasting trial at 2 h post-lunch (breakfast trial, 5·99 (sd 0·75) mm *v.* fasting trial, 6·49 (sd 1·02) mm; *P*= 0·06) but no difference between trials 3 h after lunch (*P*= 0·6).

### Insulin

There were main effects of time, trial and a trial × time interaction for serum insulin concentrations (all *P <*0·01). Following breakfast consumption, insulin concentrations were significantly greater during the morning than during the fasting trial (all *P <*0·01, Fig. 2(b)). Following lunch, insulin concentrations were significantly greater in the morning fasting trial than in the breakfast trial (all *P <*0·01).

### NEFA

There were main effects of time, trial and a trial × time interaction for plasma NEFA concentrations (all *P <*0·01). NEFA concentrations were suppressed following breakfast consumption throughout the day but remained elevated in the morning fasting trial prior to lunch (all *P <*0·01, Fig. 2(c)). Following lunch, NEFA concentrations were strongly suppressed by feeding in the morning fasting trial such that there was no difference between the trials (all *P>*0·05).

### Acylated and total ghrelin

**Fig. 3:**
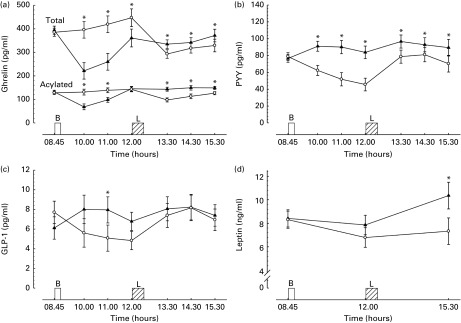
Hormonal responses during trials. (a) Plasma acylated and total ghrelin (*n* 32), (b) plasma peptide tyrosine–tyrosine (PYY, *n* 32), (c) plasma glucagon-like peptide-1 (GLP-1, *n* 32), (d) serum leptin (*n* 32), where missing data are due to insufficient blood for analysis. Values are means with their normalised CI represented by vertical bars. * Mean value was significantly different from the corresponding time point in other trial (*P*< 0·05). B, breakfast period, in which participants ate a prescribed breakfast during the breakfast trial and rested during the morning fasting trial. L, *ad libitum* pasta lunch. –▲–, Breakfast; –○–, fasting.

There were main effects of time and a trial × time interaction for plasma acylated ghrelin (both *P <*0·01). Breakfast consumption suppressed acylated ghrelin concentrations such that these were significantly lower 1 and 2 h post-breakfast relative to the morning fasting trial (both *P <*0·01, Fig. 3(a)), but there was no difference between trials 3 h post-breakfast. Acylated ghrelin concentrations were significantly greater than in the morning fasting trial throughout the afternoon in the breakfast trial (all *P <*0·01).

There were main effects of time, trial and a trial × time interaction for plasma total ghrelin concentrations (all *P <*0·01). Following breakfast consumption, total ghrelin concentrations were suppressed, resulting in significantly lower concentrations than in the morning fasting trial prior to lunch (all *P <*0·01, Fig. 3(a)). Following lunch, there was greater suppression of total ghrelin in the morning fasting trial, such that the concentrations were significantly lower than in the breakfast trial throughout the afternoon (all *P*≤ 0·05).

### Peptide tyrosine–tyrosine

There were main effects of time, trial and a trial × time interaction for plasma PYY concentrations (all *P <*0·01). Greater PYY concentrations throughout the day following breakfast consumption were apparent (*P <*0·05, Fig. 3(b)).

### Glucagon-like peptide-1

There was a tendency for a main effect of time (*F*= 2·27, *P*= 0·08), but no effect of trial (*F*= 2·89, *P*= 0·1) for plasma glucagon-like peptide-1 concentrations. There was a trial × time interaction (*F*= 3·09, *P*= 0·03) for glucagon-like peptide-1, with greater concentrations observed at 2 h after breakfast consumption when compared with the morning fasting trial but no differences between trials after lunch (Fig. 3(c)).

### Leptin

Serum leptin concentrations in both trials are displayed in Fig. 3(d). There were main effects of time, trial and a trial × time interaction for leptin (all *P <*0·02). In the breakfast trial, leptin concentrations were reduced from baseline at 3 h post-breakfast, but this reduction was greater in the morning fasting trial, such that there was a trend for lower leptin concentrations (breakfast trial, 7·86 (sd 7·28) ng/ml *v.* fasting trial, 6·79 (sd 6·63) ng/ml; *P*= 0·07). Three hours post-lunch, leptin concentrations had increased in the breakfast trial to a greater extent than in the morning fasting trial, resulting in significantly greater concentrations of leptin in the breakfast trial (*P <*0·01).

### Subjective appetite ratings

**Fig. 4:**
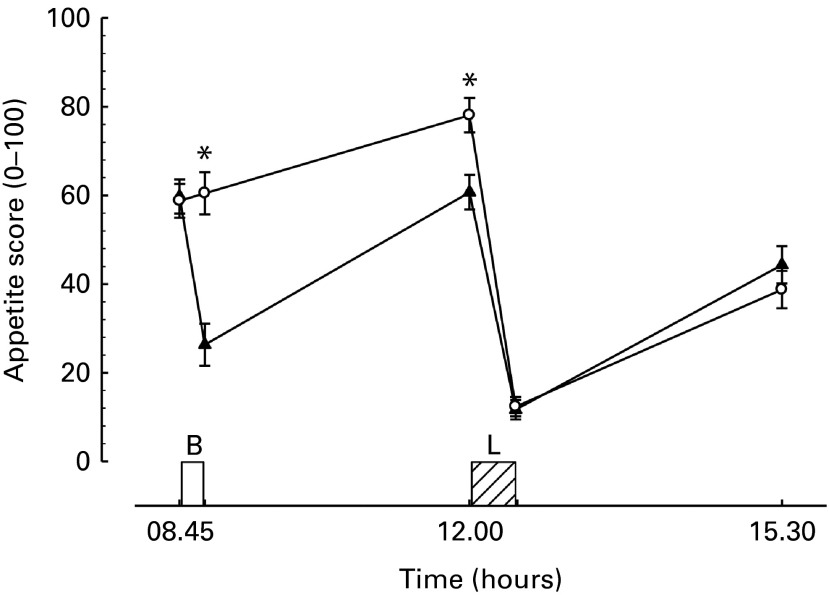
Appetite score during trials. B, breakfast period, in which participants ate a prescribed breakfast during the breakfast trial and rested during the morning fasting trial. L, *ad libitum* pasta lunch. *n* 34, as one individual was not provided with hedonic scales on one of their trials. Values are means with their normalised CI represented by vertical bars. * Mean value was significantly different from the corresponding time point in other trial (*P*< 0·01). –▲–, Breakfast; –○–, fasting.

The composite appetite score combining the hedonics obtained is displayed in Fig. 4. Desire to eat, hunger and prospective consumption all followed very similar patterns throughout the day. Immediately after breakfast consumption, there was a reduction in all three measures such that there were significant differences between all three measures compared with the morning fasting trial (all *P <*0·01, data not shown). Immediately prior to lunch, these appetite sensations remained greater in the morning fasting than in the breakfast trial (all *P <*0·01). Following the consumption of the *ad libitum* lunch, all three measures decreased to a nadir with no significant difference between trials (all *P>*0·4). Three hours after completion of lunch, there was no difference between trials for any of the measures (all *P*≥ 0·1). The sensations of fullness followed a similar, but opposite, pattern throughout the day, with fullness significantly greater after breakfast and prior to lunch in the breakfast trial (both *P*< 0·01) but not different between trials at any other time point (all *P*>0·2).

## Discussion

The present study investigated EI along with hormonal, metabolic and subjective appetite responses to an *ad libitum* lunch in an overnight fasted state in contrast with following a standardised breakfast in lean individuals. EI at the *ad libitum* lunch was greater following morning fasting than breakfast. However, in accordance with our hypothesis, greater lunch intake was insufficient to compensate for energy from breakfast, resulting in lower net intake in the morning fasting trial. Following lunch, the response of hormones associated with satiety (PYY, leptin) was greater in the breakfast trial but, paradoxically, acylated/total ghrelin were not suppressed after lunch in the breakfast trial. Suppression of ghrelin would be expected due to the substantial energy/carbohydrate intake at lunch. In contrast to our hypothesis, subjective appetite 3 h after lunch was not different between trials; indicating that morning fasting may not cause greater appetite than breakfast in the afternoon despite incomplete compensation at lunch and lower concentrations of satiety-inducing hormones. This may be due to abolished ghrelin suppression after lunch following breakfast.

PYY has been shown to reduce food intake^(^ ^20^ ^)^ with increased caloric load increasing concentrations^(^ ^38^ ^)^. In the present study, PYY increased in response to breakfast, remaining higher than the morning fasting trial throughout the day, despite greater intake at lunch following morning fasting. This is consistent with the accumulated difference in intake, and is supported by a report of similar PYY concentrations between conditions after an *ad libitum* lunch at which participants ate sufficient to compensate energetically for an omitted breakfast^(^ ^17^ ^)^. In combination, this suggests PYY is more a reflection of nutritional status over the entire day rather than in response to the most recent feeding, consistent with PYY peaking 1–2 h after feeding, followed by an elevation lasting several hours^(^ ^43^ ^)^.

Leptin concentrations were also greater 3 h after lunch in the breakfast trial. This finding is in accord with the slow response of leptin to feeding, such that increased concentrations relative to the morning fasting trial approximately 6 h after breakfast are likely a product of postprandial glucose and insulin responses to breakfast and lunch^(^ ^44^ ^)^. It is possible that the diurnal phase of leptin may have shifted in the morning fasting trial such that the usual increase later in the day was delayed. Indeed, postponing food intake from 07.00 to 13.30 hours has been shown to shift the rise in leptin later in the day^(^ ^45^ ^)^.

The greater PYY and leptin during the afternoon in the breakfast trial would be expected to contribute to greater satiety at the end of the trial, although there was no difference in subjective appetite between the trials 3 h after lunch. This may be due to the complete lack of ghrelin suppression following lunch in the breakfast trial. This is a particularly unusual result that, to our knowledge, has not been reported previously in lean individuals. As increasing energy content of liquid breakfasts with carbohydrate causes greater reductions in ghrelin^(^ ^46^ ^)^, it is particularly puzzling that the *ad libitum* lunch had no impact on ghrelin as the lunch during the breakfast trial was 3247 (sd 1460) kJ (776 (sd 349) kcal) and rich in carbohydrate. Ghrelin has been associated with meal initiation^(^ ^18^ ^)^ and as such it would be unexpected for this hormone to remain elevated after the second meal of the day. Foster-Schubert *et al.* ^(^ ^47^ ^)^ have shown that an 80 % carbohydrate breakfast drink (providing 20 % of daily energy requirements) suppressed ghrelin initially, but concentrations subsequently rebounded above fasting concentrations after 3 h, remaining elevated for the majority of the next 3 h. An obvious distinction presently is that 3 h after initial feeding, the participants received another carbohydrate-rich meal. This indicates that in the breakfast trial the ghrelin response to the second meal was abolished, with ghrelin following the established time course of a similar carbohydrate load without a second feeding occasion.

There have been reports that insulin may play an important role in ghrelin suppression^(^ ^46^ ^,^ ^48^ ^–^ ^50^ ^)^, although this view is not universal^(^ ^51^ ^,^ ^52^ ^)^, with some authors suggesting that food intake specifically suppresses ghrelin but not the administration of insulin^(^ ^51^ ^)^. It is interesting to note that when consuming breakfast, insulin responses were significantly diminished after lunch relative to the morning fasting trial. It is conceivable the substantial reduction in insulin concentrations during the afternoon may have contributed to the absence of ghrelin suppression after lunch in the breakfast trial. However, this lack of suppression needs confirmation, and it remains to be seen whether elevated ghrelin following a lunchtime meal as observed translates to greater intake throughout the rest of the day. It has been suggested in a time-blinded study that ghrelin needs to reach a ‘threshold’ (reported as 93 % of fasting concentrations) prior to meal requests^(^ ^53^ ^)^. As ghrelin in the morning fasting trial had returned to baseline concentrations 3 h after lunch (the point at which appetite was assessed in the afternoon), this may explain why there was no difference in appetite detected despite greater concentrations of ghrelin in the breakfast trial. In addition, as previously discussed, although ghrelin was greater throughout the afternoon, anorectic hormones were also elevated in the breakfast trial. Finally, it may be that ghrelin concentrations following repeated meals may not be as relevant in signalling hunger as prior to the first/second meal of the day.

Whilst reduced insulin and glucose concentrations after lunch may be partly explained by the slight reduction 640 (sd 1042) kJ (153 (sd 249) kcal) in lunch intake following breakfast, this is also potentially representative of a second meal effect on glucose^(^ ^54^ ^)^, with an associated reduction of insulin concentration and potentiation of its effects^(^ ^55^ ^)^. Evidence that reduced insulin concentrations after lunch were due to the prior meal and not just reduced intake is demonstrated in the eleven individuals who ate similar lunches in both trials (i.e. more in the breakfast trial or intake within 335 kJ (80 kcal) in both trials). Ten of these individuals had reduced insulin concentrations 60 min after lunch during the breakfast trial, with concentrations 68 (sd 25) % of the morning fasting trial, providing evidence of acutely reduced insulin sensitivity following extended fasting.

Previous studies both support^(^ ^17^ ^)^ and oppose the possibility that breakfast omission is compensated for at lunch^(^ ^15^ ^)^, with both studies including a mid-morning preload (i.e. a fixed feeding occasion 90 min before lunch). When this ‘snack’ feeding prior to lunch is removed and the fast remains unbroken until the *ad libitum* lunch, Levitsky & Pacanowski^(^ ^16^ ^)^ report that intake at lunch was either unaffected, or was reduced by 728 kJ (approximately 174 kcal), following prescribed 1464 kJ (350 kcal) breakfasts or a self-selected 2611 kJ (approximately 624 kcal) buffet breakfast, respectively. Lunch intake in the present study was only reduced to a similar degree as the larger breakfast in the aforementioned study. Therefore, it appears that any persistent satiating effect of a typical breakfast at lunch is only brought about by breakfast intake of a magnitude that cannot reasonably be matched solely by reduced intake at lunch.

A limitation of the present study is that without intake data for the remainder of the day after lunch, it is not possible to establish whether further energetic compensation would occur within the 24-h period. However, laboratory work measuring intake following lunch through the afternoon/evening (including snacking opportunities) has not identified greater consumption at any feeding occasion following lunch after morning fasting^(^ ^16^ ^)^. Limited daily compensation has also been observed when fasting through the morning during free living^(^ ^25^ ^,^ ^56^ ^)^. Another potential limitation is that, whilst each individual participant repeated dietary intake for 48 h prior to each laboratory visit, this pre-trial diet was not standardised between participants. While any variance between different individuals would not confound the overall interpretation between trials within this repeated measures design, it could contribute to greater variability in inter-individual responsiveness between treatments, given that an extreme shift in macronutrient composition of an evening meal (i.e. 16 *v*. 62 % energy as fat) can affect next-day metabolic outcomes^(^ ^57^ ^)^. However, an additional consideration is that a standardised evening meal would not represent habitual intake for each individual. It should also be noted that appetite sensations were obtained relatively infrequently and this may have reduced our ability to detect differences between trials during the afternoon.

Future laboratory studies should include further feeding opportunities after lunch while extending metabolic and hormonal measures through the evening to elucidate the role of these physiological responses in influencing feeding throughout the duration of the day after morning fasting. As the first study to report the unexpected response of ghrelin to repeated carbohydrate-rich/high glycaemic index feedings, it is important for further research to replicate these findings and also examine breakfasts of different macronutrient composition and glycaemic index. In addition, due to the suggested appetite suppressive effects of higher protein breakfasts in adolescents^(^ ^19^ ^,^ ^21^ ^)^ and during the morning in adults^(^ ^58^ ^)^, the effects of sequential meals on metabolic and hormonal responses following high protein breakfasts should be further examined in adults.

In summary, while morning fasting was incompletely compensated for at an *ad libitum* lunch, prior carbohydrate-rich breakfast consumption increased concentrations of some satiety hormones after lunch but abolished suppression of the orexigenic hormone ghrelin. This novel finding may be mediated through reduced insulin response to a second meal and results in similar appetite during the afternoon independent of morning feeding pattern.
